# Antimicrobial Polymer-Based Materials for Food Packaging Applications

**DOI:** 10.3390/polym12040731

**Published:** 2020-03-25

**Authors:** Ana María Díez-Pascual

**Affiliations:** Department of Analytical Chemistry, Physical Chemistry and Chemical Engineering, Faculty of Sciences, Institute of Chemistry Research “Andrés M. del Río” (IQAR), University of Alcalá, Ctra. Madrid-Barcelona, Km. 33.6, 28871 Alcalá de Henares, Madrid, Spain; am.diez@uah.es; Tel.: +34-918-856-430

Antimicrobial packaging has recently attracted a great deal of interest from the food industry due to the boost in consumer demand for minimally-processed, preservative-free products. Antimicrobial polymeric packaging systems can be considered an emerging technology that could have an important impact on shelf life extension and food safety. Novel polymeric-based packaging materials are continually being developed. Multifunctional composites with desired properties can be tailored by combining reinforcement phases with antimicrobial agents into a polymer matrix. Thus, the importance of active agents like metal nanoparticles, essential oils, or natural extracts in different polymeric matrices has been demonstrated in a broad number of papers [1,2]. One of the main ideas behind this approach is to improve the matrix barrier and mechanical performance. In particular, the use of nanoparticles instead of conventional fillers is beneficial from an application viewpoint, given that the nanoscale fillers display higher specific surface area and density compared to microparticles; therefore, lower nanofiller concentrations are required to reach properties equivalent to or even better than those obtained by conventional microfiller loadings, which makes the processing easier and minimizes the raise in composite weight. Nonetheless, although the aim of these new materials is to improve packaged food quality and safety, the toxicological effects derived from their potential migration from the polymer structures is still under consideration. This Special Issue, with a collection of 12 original contributions and one review, provides selected examples of the most recent advances in the preparation and characterization of antimicrobial composites for food packaging applications.

Amongst the major reasons for food deterioration and spoilage are the presence of foodborne pathogens and other microorganisms, including bacteria such as *Campylobacter jejuni*, *Clostridium botulinum*, *Escherichia coli*, *Listeria monocytogenes*, *Salmonella* spp., *Staphylococcus aureus*, *Vibrio* spp. and *Yersinia enterocolitica*, viruses like *Hepatitis A* and Noroviruses and parasites such as *Cyclospora cayetanensis*, *Toxoplasma gondii* and *Trichinella spiralis* [3]. Consequently, the demand for antibacterial drugs in food packaging is continuously rising. In this regard, Huang et al. [4] have reviewed the different types of biodegradable and non-biodegradable materials used for food packaging and the most important antibacterial agents, both natural and synthetic, employed to restrain the growth of bacteria. Synthetic ones, like ethylenediaminetetraacetic acid (EDTA) and fungicides, are advantageous since they are more economic and present better activity, though they usually exhibit higher toxicity. Conversely, natural antimicrobials obtained from materials in nature, like essential oils (EOs), bacteriocins, lysozyme, chitosan, acid compounds (i.e., ascorbic acid, citric acid, lactic acid) and grape fruit seed extracts (containing numerous phenolic compounds such as catechins, epicatechin, gallic acid and procyanidins), frequently require complex extraction procedures. 

EOs, also known as volatile oils, since they contain volatile chemical compounds extracted from plant materials such as flowers, buds, seeds, leaves, wood, fruits, roots, barks, etc., possess antibacterial, antifungal, antioxidant, antibiotic and antiseptic properties [5]. They can be used as antimicrobial agents by mixing with the basic materials, by coating onto the food packages or by loading into an antibacterial pouch. Among these approaches, mixing EOs with other polymers is frequently used since it is suitable and easily applied to a large scale.

On the other hand, bacteriocins such as nisin and pediocin are bactericidal substances generated by bacteria, encoded by genes and synthesized by ribosomes, which can be used as antimicrobial agents [6]. Lysozyme, a hydrophilic monopeptide chain, can also restrain bacterial infections, particularly those induced by Gram-positive bacteria. Its antimicrobial action is owed to its ability to hydrolyze the β-1–4 glycosidic bonds between N-acetylmuramic acid and N-acetylglucosamine in peptidoglycans [7], the main cell wall component, demolishing the cell wall, thus provoking intracellular materials to leak out and leading to bacterial death. Grape fruit seed extracts also display a large range of microbial growth inhibition against both Gram-positive and Gram-negative bacteria [8], together with antiseptic, germicidal, antibacterial, fungicidal and antiviral properties.

Chitosan (CS) is a biopolymer with good film-forming ability and intrinsic antimicrobial properties widely applied in the fields of biomedicine, food packaging and environmental protection [9]. It is produced from chitin with a linear structure, which is constituted by random deacetylated unit and acetylated unit. CS antimicrobial activity depends on a number of factors, including its molecular weight, degree of deacetylation, degree of substitution, physical form, as well as structural properties of the cell wall of the target microorganisms [10]. The antimicrobial action of CS has been attributed to different mechanisms (Figure 1) [11,12]: (I) Electrostatic attractions between its positively-charged chains and the negatively-charged bacterial cell walls, causing the polymer absorption onto the target bacteria, leading to cell wall disruption. (II) Generation of reactive oxygen species (ROS). (III) Its chelating effect on metals and oligoelements, which are essential for bacterial growth, and their deficiency results in bacterial death. Among CS disadvantages are its rapid dissolution in acidic solutions and poor mechanical and processing properties, making it difficult to be processed into food packaging. The electrospinning of chitosan in the form of nanofibers is another promising process that has been widely explored [13].

Layered double hydroxides (LDHs) comprise positively-charged brucite-like layers of divalent and trivalent metal hydroxides, in which the excess of positive charges is compensated by anions and water molecules present in the interstitial position [14]. They show important properties, such as biocompatibility, null toxicity, and allergenicity. In particular, ZnAl hydroxides are effective antimicrobial agents for *E. coli* and *S. aureus* bacteria. ZnAl layered double hydroxide-CS hybrids have been prepared by mixing Zn/Al molar ratio of 5.0 in deionized water and adding CS concentrations in the range 0–3.0 g∙L^−1^ [15]. The mixture was placed into a three-necked round-bottomed flask, and urea was added (urea/NO3− molar ratio of 4:1). The structure and surface properties of the hybrids were characterized by X-ray diffraction (XRD), Fourier-transform infrared spectroscopy (FTIR), scanning electron microscopy (SEM), UV-Vis spectroscopy and zero point charge techniques. CS amount is a major factor influencing the antibacterial activity of the hybrids. An increase in the antibacterial activity was found for CS concentrations in the range of 0.5 to 1.5 g L^−1^, while higher loadings led to poorer antimicrobial activity, since the high CS content disrupts the antibacterial action of the metallic nanostructures released from LDH.

Amongst the non-biodegradable polymers used in antimicrobial food packaging are polypropylene (PP), high-density polyethylene (HDPE), low-density polyethylene (LDPE), poly(vinyl chloride) (PVC), poly(ethylene-co-vinylacetate) (EVA) and poly(ethylene terephthalate) (PET). However, nowadays it is increasing the use of biodegradable polymers such as polylactic acid (PLA), cellulose, starch and CS, particularly in countries where landfills are the major way of waste management, because they are green, sustainable and environmentally friendly. PLA resulting from renewable substances such as potato, wheat or corn starch is biodegradable, and is regarded as a safe (GRAS) substance. It shows a large number of advantages including non-carcinogenicity, biocompatibility, sustainability, hydrophilicity, water solubility and chemical stability. However, it is highly permeable to gas and vapor, which limits its use for short-life packaged food [16]. Therefore, it is usually mixed with other polymers like cellulose, polycaprolactone (PCL) and polyhydroxybutyrate (PHB). Further, in order to develop antimicrobial properties [17], it can be mixed with plant extracts (e.g., lemon), essential oils (e.g., carvacrol and thymol, present in oregano oil), enzymes (e.g., lysozyme) and metals (e.g., silver).

In this regard, the antimicrobial properties and water vapor performance of PLA films coated with a cellulose derivative/cocoa butter carrier incorporating *Eucomis comosa* extract as an active substance have been investigated [18]. The coatings reduced the water vapor permeability of the biopolymer and provided improved resistance against UV-aging, which is desirable to extend the shelf life, quality and freshness of food products. 

PLA packages have also been filled with bioactive molecules derived from EOs such as carvacrol and thymol [19]. In order to reduce the high volatility and reactivity of these compounds, they have been encapsulated by cyclodextrins (CDs), cyclic oligosaccharides made of 6 (α), 7 (β), or 8 (γ) units of D-glucose monomers linked by α(1,4) bonds, that have an internal hydrophobic cavity suitable to interact with bioactive molecules of EOs, while the external part is hydrophilic, improving their water solubility and steadily increasing their effectiveness at low concentrations. Different loadings in the range of 0 to 5 wt.% of β-CD–thymol or β-CD–carvacrol were mixed with PLA via injection process, and their mechanical, structural and thermal properties were investigated. The polymer Young´s modulus and tensile strength were significantly reduced with increasing content of β-CD–thymol or β-CD–carvacrol due to their plasticizing effect onto the polymer matrix, disrupting its crystalline structure and increasing its ductile properties, which is beneficial in order to avoid breakages during processing. Further, thermogravimetric analysis (TGA) showed a small drop in the temperature of degradation of the package as the concentration of the complexes increased. More importantly, packages containing 2.5% and 5% β-CD–carvacrol or 5% β-CD–thymol showed inhibition against *Alternaria alternata* upon 10 days of incubation, which is interesting from both the economic and social viewpoints, since Alternaria fungi are among the main pathogens causing post-harvest diseases and significant economic losses. Therefore, these novel biopackages have could be used in the agro-food industry.

PLA-based composites filled with different contents of TiO_2_ nanoparticles (0–20 wt.%) have been prepared by solvent casting followed by hot-pres processing [20]. Structural characterization carried out by X-ray diffraction (XRD) and FTIR spectroscopy did not show modifications in the polymer structure due to the presence of the nanoparticles, while TGA demonstrated a small rise in the degradation temperature with increasing nanoparticle content. The presence of the nanoparticles inhibits *E. coli* growth and biofilm development, ascribed to a direct interference on bacterial metabolism. 

Active nanocomposite packaging films based on PLA reinforced with nano-Ag were prepared via solvent evaporation method and used as packaging for strawberries [21]. Compared with pristine PLA, the nanocomposites had better physical properties, and successfully reduced the weight loss rate of strawberries during storage, and delayed the reduction in hardness, soluble solids and titratable acid content. In particular, the active packaging film with 5 wt.% nano-Ag showed the best preservation effect of the strawberries freshness. 

On the other hand, cellulose, a polysaccharide consisting of a linear chain of β(1→4) linked D-glucose units, is the most abundant natural polymer, which can be extracted from corncobs. It is environmentally friendly and biodegradable. Composite films made of cellulose and its derivatives such as methylcellulose (MC), hydroxypropylmethylcellulose (HPMC) and carboxymethylcellulose (CMC) are currently attracting a lot of interest to produce films due to their suitable properties, recyclability and degradability. However, their high cost and high water permeability restrict their applications. In this regard, bacterial cellulose has been mixed with CS cross-linked with borate, tripolyphosphate, or their mixture, and composite films were fabricated via solution casting. The cross-linking increased the mechanical properties, such as the tensile strength, and composites crosslinked with the borate/tripolyphosphate mixture exhibited the best properties. However, the antibacterial activity was reduced compared to non-crosslinked composites [22].

With the aim to design new biomaterials via eco-friendly processes, bacterial cellulose nanofibers have been mixed with PHB by a melt compounding technique followed by plasma treatment [23]. Besides, to attain improved antibacterial activity, ternary nanocomposites incorporating a plasma coating made of ZnO nanoparticles have been developed. The plasma treatment maintained the thermal stability, crystallinity and melting behavior of the binary nanocomposites, despite their nanofiber content, while it increased the mechanical performance and antimicrobial activity. More importantly, the ZnO plasma coating totally restricted the growth of Gram-positive bacteria, hence the ternary composites are great candidates as green food packages.

Besides cellulose and hemicellulose, lignin attracts scientific interest as a source of aromatic compounds, representing 30% of all non-fossil organic carbon on Earth. In addition to its abundance and inexpensive supply, it displays many attractive properties, such as biodegradability, antioxidant activity (due to its phenolic structure), high carbon content, high thermal stability, and stiffness [24]. HPMC/lignin and HPMC/lignin/CS films with HPMC contents in the range of 1–30 wt.% have been prepared by solution processing [25]. Efficient antimicrobial activities against both Gram-positive and Gram-negative bacteria were found at both 35 °C and low (0–7 °C) temperatures. Storage resulted in an increase in antimicrobial activity against Gram-positive bacteria due to the degradation of lignin over time. The scavenging and antimicrobial activities of both binary (HPMC/lignin) and ternary (HPMC/lignin/CS) composites were influenced by the lignin concentration: The composite with 5% loading showed the best activity and the 30% the poorest.

Starch, a polysaccharide comprising glucose monomers joined in α(1→4) linkages, can also be used as a food packaging material, acting as an adhesive, additive or thickener. It behaves as a moderate oil barrier, while it absorbs the moist from the environment due to its large amount of hydrophilic groups [26]. To improve its chemical, mechanical and barrier properties, different components such as blackberry pulp microparticles synthesized via freeze-drying can be added [27]. The incorporation of these antioxidant particles makes the starch surface rougher, thicker, more flexible and more soluble in water, while it reduces its mechanical strength. The performance of the films was conditioned by the particle loading and its way of incorporation in the matrix (direct or by sprinkling). Nanocomposites with 20 wt.% loading displayed reduced water vapor permeability compared to the starch matrix, due to the improved dispersion of the nanoparticles within the matrix and the formation of tortuous paths for water diffusion. However, higher contents resulted in higher water vapor permeability owed to the formation of particle aggregates. Nanocomposites with particles introduced by sprinkling had higher antioxidant capacity and water solubility, hence higher potential to release bioactive compounds when used in food packaging materials.

With regard to the EOs, a lot of research has been devoted over the last years to incorporate them into food packages. EOs contain a variety of substances, such as terpenes and phenolic compounds, which are responsible for their antibacterial properties. They have recognized a GRAS status and are the most widely used additives in the food industry to provide antimicrobial function. The EO derived from cinnamon bark contains about 50% cinnamaldehyde, an effective antimicrobial fungicide and insecticide. The addition of cinnamon active chemicals to packaging systems significantly reduces the growth rate of microorganisms [28], thus increasing the life-span. For such purpose, cinnamon oil was added to algae films, and the resulting compounds were characterized by soil bury tests, tensile tests, FTIR and SEM [29]. The best mechanical performance and longest shelf-life rate of the films was attained upon addition of 5 wt.% cinnamon. 

Another attractive EO is orange oil, which is typically extracted as a by-product of orange juice production by centrifugation. It is composed mostly of terpenes, in particular d-limonene, and long-chain aliphatic hydrocarbon alcohols and aldehydes. The incorporation of low loadings (i.e., 3 wt.%) of orange oil into mango peel pectin film strongly improves the antimicrobial action against Gram-positive bacteria, hence it is a very suitable candidate as antibacterial material for food packaging [30].

The incorporation of EOs into polymers provides important advantages in terms of their wide-spectrum activity and safety. Further, since they can be released as a vapor, their antimicrobial role does not entail direct contact with the target microorganism. However, the integration of EOs into commodity polymers using traditional manufacturing processes is a challenging task due to their loss during high-temperature processing and reduced antimicrobial efficacy [31]. Halloysite nanotubes (HNTs), naturally-occurring clays with a tubular structure and chemical composition similar to kaolin [32], can act as active carriers for sensitive oils such as carvacrol, present in the oil of thyme (obtained from pepperwort and wild bergamot). This is an effective strategy to incorporate EOs into plastic polymers without lost in antimicrobial function [33]. The HNTs/carvacrol mixtures hybrids can be melt-compounded with synthetic polymers such as LDPE to fabricate ternary LDPE/(HNTs/carvacrol) composites, followed by a multilayer coextrusion process at high temperature with an ethylene vinyl alcohol copolymer (EVOH). The resulting multilayered (LDPE/[HNTs/carvacrol])/EVOH films showed tailored structure and thickness from the micro to the nanoscale, and displayed excellent antimicrobial action against Gram-negative bacteria and fungi like *A. alternata* and Rhizopus. This novel approach provides the ability to customize packaging for a variety of food products that are affected by bacteria, such as meat and fish, or by molds such as cheese, bread and fresh products.

Thermosetting polymers derived from EOs also display a wide range of outstanding properties that make them suitable for food packaging [34]. Even so, the antibacterial properties of these materials can be considerably improved by addition of very small amounts of metal oxide nanoparticles such as TiO_2_ [35] or ZnO [36]. The antimicrobial efficiency of these nanoparticles is influenced by their size, shape, concentration and degree of functionalization [37,38]. Due to their nanoscale size, very high specific surface area and controllable surface chemistry, the nanoparticles can distinguish between bacterial cells and mammalian ones [39]. Their antimicrobial function has been attributed to the generation of ROS that can target physical structures, metabolic paths, as well as DNA synthesis. Other mechanisms of action like lipid peroxidation, cell membrane lysis, redox reactions at the nanoparticle–cell interface, bacterial phagocytosis, etc. have also been reported [40].

## Figures and Tables

**Figure 1:**
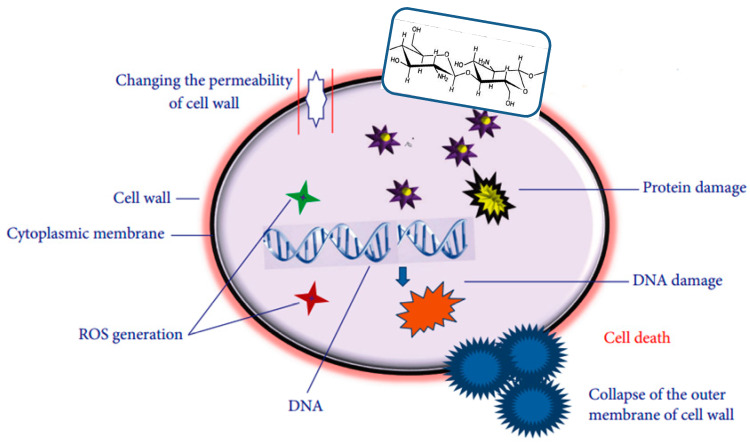
Principal mechanisms of antimicrobial action of chitosan. Adapted from [11], copyright © 2020, Xing et al.
